# Impact of Co-Ensiling of Maize with *Moringa oleifera* on the Production of Greenhouse Gases and the Characteristics of Fermentation in Ruminants

**DOI:** 10.3390/ani13040764

**Published:** 2023-02-20

**Authors:** Edwin Rafael Alvarado-Ramírez, Aristide Maggiolino, Mona M. M. Y. Elghandour, Marco Antonio Rivas-Jacobo, Gilberto Ballesteros-Rodea, Pasquale De Palo, Abdelfattah Z. M. Salem

**Affiliations:** 1 Faculty of Agronomy and Veterinary, Autonomous University of San Luis Potosí, 78321 San Luis Potosí, Mexico; 2 Department of Veterinary Medicine, University of Bari A. Moro, 70010 Bari, Italy; 3 Faculty of Veterinary Medicine and Zootechnics, Autonomous University of the State of Mexico, 50000 Toluca, Mexico

**Keywords:** carbon monoxide, hydrogen sulfide, methane, ruminal fermentation, ruminants, *Zea mays* L.

## Abstract

**Simple Summary:**

The mixture of maize (*Zea mays* L.) with *Moringa oleifera* (MOL) during silage can improve the nutritional quality of the silage, but the impact of the proportion of both forages on fermentation and rumen production of greenhouse gases has not been assessed and evaluated. Therefore, the objective of this experiment was to evaluate the impact of maize co-ensiling with increasing percentages of MOL forage on the kinetics of biogas, methane (CH_4_), carbon monoxide (CO), and hydrogen sulfide (H_2_S) production, as well as the characteristics of ruminal fermentation and CH_4_ conversion efficiency, using steers and sheep as inoculum sources. The results indicated that the co-silage of maize with MOL improved the degradability with both sources of inoculum, and that regardless of the percentage of MOL, the steer inoculum presented the highest values in the biogas production, CH_4_, H_2_S, degradability of dry matter, short-chain fatty acids (SCFA), and metabolizable energy (ME), as well as the lowest pH and highest CH_4_ conversion efficiency, in terms of CH_4_ produced per unit of SCFA, ME, and organic matter.

**Abstract:**

The objective of this experiment was to evaluate the impact of maize co-ensiling with increasing percentages of MOL forage on the kinetics of biogas, methane (CH_4_), carbon monoxide (CO) and hydrogen sulfide (H_2_S) production, as well as the characteristics of ruminal fermentation and CH_4_ conversion efficiency, using steers (STI) and sheep (SHI) as inoculum sources. With the STI, the inclusion of MOL reduced (linear: *p* ≤ 0.0199; quadratic: *p* ≤ 0.0267) biogas production (mL g^−1^ DM incubated and degraded), CH_4_ (mL g^−1^ DM degraded), CO (mL g^−1^ DM degraded), and H_2_S (mL g^−1^ DM incubated and degraded), without affecting (*p* > 0.05) the parameters (*b* = asymptotic gas, *c* = rate of gas production and *Lag* = initial delay time before gas production) of CH_4_ and H_2_S, and the proportion and production of CH_4_ per kg of dry matter (DM). In addition, with this inoculum, pH, and dry matter degradation (DMD) increased (linear: *p* ≤ 0.0060), and although short-chain fatty acids (SCFA) and metabolizable energy (ME) decreased (linear: *p* < 0.0001; quadratic: *p* ≤ 0.0015), this did not affect (*p* > 0.05) the CH_4_ conversion efficiency. Meanwhile, with the SHI, the inclusion of MOL only decreased (linear: *p* ≤ 0.0206; quadratic: *p* ≤ 0.0003) biogas per dry matter (DM) degraded and increased (linear: *p* ≤ 0.0293; quadratic: *p* ≤ 0.0325) biogas per DM incubated, as well as the production (mL g^−1^ DM incubated and degraded and g^−1^ kg DM) and proportion of CH_4_, and CO per DM incubated and degraded. In addition, it did not impact (*p* > 0.05) on the CH_4_ and H_2_S parameters, and in the H_2_S by DM incubated and degraded, and although it increased (linear: *p* ≤ 0.0292; quadratic: *p* ≤ 0.0325) the DMD, SCFA, and ME, it was inefficient (quadratic: *p* ≤ 0.0041) in CH_4_ conversion. It is concluded that regardless of the percentage of MOL, the STI presented the highest values in the production of biogas, CH_4_, H_2_S, DMD, SCFA, and ME, and the lowest pH, so it turned out to be the most efficient in CH_4_ conversion, while with the SHI only the highest production of CO and pH was obtained, and the lowest DMD, SCFA, and ME, so it was less efficient compared to STI.

## 1. Introduction

Livestock is an activity that contributes significantly to climate change [1], especially ruminant cattle due to the emission of enteric biogas into the environment, since it is mainly made up of carbon dioxide (CO_2_; 45–70%), methane (CH_4_; 20–30%), and in smaller quantity by oxygen (O_2_), hydrogen (H_2_), carbon monoxide (CO), hydrogen sulfide (H_2_S), among other gases [2]. Of these gases, CH_4_ and CO_2_, when released into the atmosphere, exert a greenhouse effect, thus favoring the increase in global warming [3], and although their production is inevitable since they result from ruminal fermentation of the feed [4,5], high amounts reflect poor digestibility and a loss of metabolizable energy [6,7]. Therefore, to reduce these gases, maize silage (*Zea mays* L.) has been proposed as a strategy, since its high starch content increases the production of propionate, and this reduces the availability of H_2_ for the formation of CH_4_ [8].

As feed for ruminant livestock, maize silage has the ability to provide forage that is high in energy, starch, and fiber, but deficient in protein due to its low content [9,10]. Given this situation, the inclusion of forage of protein-rich species, such as legumes and some non-legumes, has been proposed as an option to obtain quality silage [11,12,13]. In this sense, *Moringa oleifera* (MOL) is a non-leguminous tree species native to northeastern India that is used as a low-cost feed source in livestock, since it has adapted to various agroclimatic conditions and has high nutritional value [14,15,16,17]. Therefore, it can be used to improve the quality of maize silage. It has been reported that MOL presents minerals (Ca, Fe, Zn, Mg, Mn, and Cu), high percentage of protein (30%), and digestibility (79%) [18,19,20], as well as 10 essential amino acids in animal nutrition and some secondary metabolites [21,22,23]. In the case of metabolites, it has been reported that they are capable of mitigating rumen production of greenhouse gases [24,25,26] and improving productivity [27,28,29] and animal health [30].

The chemical composition of silages can influence the rumen microbial community, and this in turn compromises the potential of maize to mitigate the production of greenhouse gases [8], so it is necessary to evaluate how it affects the percentage of each forage in silage on rumen fermentation and greenhouse gas production. The in vitro gas production technique [31] has been very useful in this type of evaluations due to its practicality and similarity with in vivo evaluations [32,33,34], in addition to the fact that a greater number of feeds can be evaluated and that it allows for the estimation of short-chain fatty acids, metabolic energy, and apparent degradability of the feed [35,36]. However, it has been reported that there are variations in fermentability patterns and in some fermentation characteristics between and within animal species, so the inoculum source turns out to be a crucial factor to consider [37,38]. Based on the above, the objective of this experiment was to evaluate the impact of co-ensiling of maize with increasing percentages of MOL forage on the kinetics of biogas, methane (CH_4_), carbon monoxide (CO) and hydrogen sulfide (H_2_S), as well as in the characteristics of rumen fermentation and CH_4_ conversion efficiency, using steers and sheep as inoculum sources.

## 2. Materials and Methods

### 2.1. Production of Forage

The forage was produced in the town of San Francisco, municipality of Victoria, Tamaulipas, Mexico (23°52′55″ N and 99°13′07″ W), at an altitude of 261 masl. The climate of the site, according to the Köppen classification, is of the Aw_1_ type, which corresponds to warm sub-humid with summer rains [39]. A native maize genotype from the State of Tamaulipas, locally known as “Olotillo” was used, and it established in August 2021, at a density of 62,500 plants ha^−1^ (0.80 × 0.20 m, distance between row and plant). The *Moringa oleifera* (MOL) forage was obtained from a 6-year-old plantation and established at a density of 40,000 plants ha^−1^ (0.50 × 0.50 m, distance between row and plant). The harvest was carried out manually in November 2021, when the maize grain reached the milky-dough state and MOL had 45 days of regrowth, and the cutting height was 10 and 25 cm above ground level, respectively. During the growth and development of the crops, fertilizers were not applied, and the weeds were controlled manually.

### 2.2. Elaboration of Micro-Silages

The forage of both crops was mechanically crushed separately until a particle size of 2 ± 1 cm was achieved, and three samples (300 g) were obtained from each one for the analysis of the chemical composition. The mixtures were made separately in the proportions of 10:0, 8:2, 6:4, 4:6, 2:8, and 0:10, corresponding to the inclusion of 0 (M0), 20 (M20), 40 (M40), 60 (M60), 80 (M80), and 100 (M100)% of MOL forage in co-ensiling with whole-plants maize. Hence, 5 kg of each mixture were taken, ensiled in triplicate in black polyethylene bags (30 cm in diameter × 50 cm in height, caliber 500), and sealed under vacuum. They were stored for 120 days at room temperature in an area free from direct solar radiation and moisture, and when they were opened, three samples (300 g) were obtained for the determination of the chemical composition and in vitro evaluation.

### 2.3. Chemical Composition

At the time of obtaining the samples, the fresh and ensiled forage underwent a dehydration process in a forced-air circulation oven at 60 °C for 72 h and was crushed in a hammer mill (Thomas Wiley^®^ Laboratory Mill model 4, Thomas Scientific™, Swedesboro, NJ, USA) with a 1-mm sieve for the determination of dry matter (DM) and chemical analysis. Organic matter (OM) was estimated by determining the percentage of ash using the method described by Maggiolino et al. [40], the crude protein (CP) multiplying the nitrogen content by 6.25 [41], and the ether extract (EE) following Padmore’s methodology [42]. The hemicellulose and cellulose were calculated by estimating the percentages of neutral detergent fiber (NDF) and acid detergent fiber (ADF) with the methodology described by Van Soest et al. [43] in the ANKOM^200^ fiber analyzer (ANKOM Technology Corp., Macedonia, NY, USA), as well as with the percentage of acid detergent lignin (ADL) calculated following the methodology proposed by Faichney and White [44]. The non-structural (NSC) and total (TC) carbohydrates were calculated according to the equations of Mertens [45] and Sniffen et al. [46] as follows:

NSC = 100 − (CP + NDF + EE + Ash)(1)

TC = 100 − (CP + EE + Ash)(2)

All estimations and calculations were made as a percentage of DM, and additionally, when opening the silages, the pH was determined using a potentiometer with a glass electrode (pH wireless electrode HALO^®^ model HI11102, Hanna^®^ Instruments, Villafranca, Padua, Italy) [47].

### 2.4. In Vitro Incubation

The incubation was carried out in glass vials with a capacity of 160 mL, and in each one 500 mg of dehydrated silage, 40 mL of nutritive solution and 10 mL of rumen liquid were placed, either from steers or sheep, as appropriate. The nutrient solution was prepared following the methodology of Goering and Van Soest [48], and the rumen fluid was obtained from the rumen content of four male steers (450 ± 25 kg LW) and four sheep (45 ± 5 kg LW) that were fed ad libitum with commercial concentrate (Purina^®^, Toluca, State of Mexico, Mexico), and with a constant supply of fresh water. These animals were sacrificed in the municipal slaughterhouse of Toluca, State of Mexico, Mexico, so the rumen content of the four animals per species was transferred separately in a hermetic thermos to the laboratory, as described by De Bellis et al. [49], where it was filtered with four layers of gauze for extraction of the ruminal liquid, and later it was mixed to generate a single sample of each species. The vials were sealed with butyl rubber stoppers and aluminum seals and incubated in an incubator at 39 °C for 48 h, but not without shaking them slightly. In total, three incubation cycles were carried out, and in each one each silage (treatment) was incubated in triplicate, in addition to three blanks (without substrate) per inoculum for the correction of the readings.

### 2.5. Ruminal Total Biogas, Methane (CH_4_), Carbon Monoxide (CO), and Hydrogen Sulfide (H_2_S)

The biogas volume was measured in PSI (pounds per square inch) at 2, 4, 6, 24, 28, 30, and 48 h of incubation, following the methodology of Theodorou et al. [50] and using a digital manometer with an accuracy of ±2% (Manometer model 407910, Extech^®^ Instruments, Nashue, NH, USA). The CH_4_, CO, and H_2_S were also quantified by the methodology proposed by Acosta et al. [49], which consists of extracting the gas from the vials with a sterile plastic syringe (BD Plastipak™, 5 mL 21 G × 32 mm) and injecting it into a portable gas detector (Dräger X-am^®^, model 2500, Dräger, Lübeck, SH, Germany) by means of an external pump (Dräger X-am^®^, Dräger, Lübeck, SH, Germany). At the end of each measurement, the gas accumulated in the upper part of the vials was released with a syringe without a plunger, to avoid a greater accumulation of gas and the partial dissolution of the gases evaluated [51].

### 2.6. Ruminal Hydrogen Potential (pH) and Dry Matter Degradability (DMD)

At the end of the incubation time, the contents of the vials were filtered using bags with 25-µm porosity (Filter bags F57, ANKOM Technology Corp., Macedon, NY, USA), the liquid was collected in beakers of glass, and pH was immediately measured using a glass electrode potentiometer (Hanna^®^ Instruments model HALO^®^ HI11102). The residues of the samples adhering to the walls of the vials were extracted by rinsing them with distilled water and collected with the bags used in the initial filtration. Once all the residues had been collected, the bags were washed with abundant tap water and subjected to a drying process at 60 °C for 72 h. From the initial weight and the residue of the sample, the apparent DMD (%) was estimated as follows:

DMD = [(SIW − SRW) / SIW] × 100(3)

where:

SIW = sample initial weight (mg)

SRW = sample residue weight (mg)

100 = conversion factor to percentage

### 2.7. Calculations

The kinetics of production of biogas, CH_4_, CO, and H_2_S were estimated by adjusting the volume of the gases with the NLIN procedure of SAS [52], according to the model proposed by France et al. [53]:

y = *b* × [1 − e^−*c* (t − *Lag*)^] (4)

where:

y = volume (mL) of biogas, CH_4_, CO and H_2_S at time t (h).

*b* = asymptotic biogas, CH_4_, CO and H_2_S production (mL g^−1^ DM).

*c* = rate biogas, CH_4_, CO and H_2_S production (gas h^−1^).

*Lag* = initial delay time before biogas, CH_4_, CO and H_2_S production begins (h).

Metabolic energy (ME; MJ kg^−1^ DM) was estimated according to the equation proposed by Menke et al. [54]:

ME = 2.20 + (0.136 × PBG) + (0.057 × CP)(5)

where:

PBG = net biogas production (mL 200 mg^−1^ DM) at 24 h of incubation.

CP = crude protein (g kg^−1^ DM).

Short chain fatty acid (SCFA; mmol 200 mg^−1^ DM) concentrations were calculated according to Getachew et al. [55]:

SCFA = (0.0222 × PBG) − 0.00425(6)

where

PBG = net biogas production (mL 200 mg^−1^ DM) at 24 h of incubation.

Additionally, the ratio between CH_4_ and the SCFA (CH_4_:SCFA; mmol mmol^−1^), ME (CH_4_:ME; g MJ^−1^) and OM (CH_4_:OM; mL g^−1^) was calculated.

### 2.8. Statistical Analysis

Before analysis, the three replicates of each treatment per incubation cycle were averaged, and the average obtained was considered as the experimental unit (EU) of each treatment. The experimental design was completely randomized with a 2 × 6 bifactorial arrangement, where factor A corresponded to the sources of rumen inoculum and factor B to the percentages of MOL, with three repetitions per treatment, which in this case corresponded to the EU. The analysis was performed with the GLM procedure of the SAS program [52], according to the following statistical model:

*Y_ijk_* = µ + *A_i_* + *B_j_* + (*A* × *B*)*_ij_* + ε*_ijk_* (7)

where *Y_ijk_* is the response variable, μ is the overall mean, *A_i_* is the effect of the inoculum source ruminal, *B_j_* is the effect of the percentage of inclusion of MOL forage, (*A* × *B*)*_ij_* the effect of the interaction between the inoculum source ruminal and the percentage of inclusion of MOL forage, and ε*_ijk_* the experimental error. Linear and quadratic effects of percentage of MOL forage were determined using orthogonal polynomial contrasts. In the comparison of means with significance, the Tukey test was applied (α = 0.05).

## 3. Results

Table 1 shows the chemical composition of the co-ensiling of whole-plants maize with different percentages of *Moringa oleifera* forage (MOL). The inclusion of MOL increased the percentage of CP, EE, LIG, and NSC, but decreased OM, HEM, and CEL, in addition to slight variations in TC.

### 3.1. Ruminal Biogas Production

**Figure 1:**
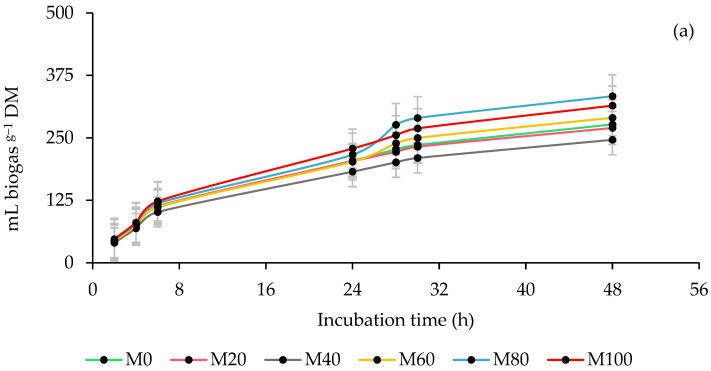

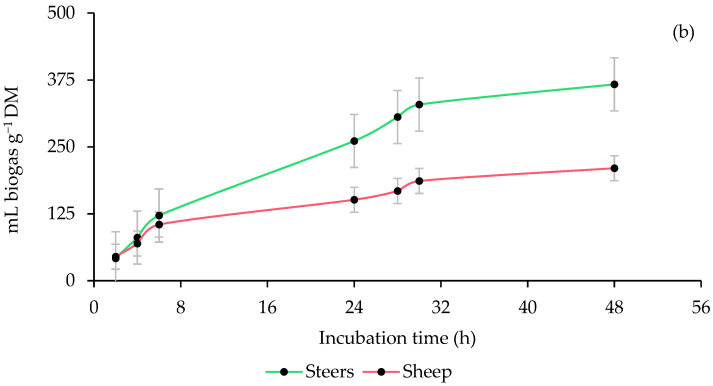
Kinetics of ruminal biogas production of co-ensiling of whole-plant maize (*Zea mays* L.) with 0 (M0), 20 (M20), 40 (M40), 60 (M60), 80 (M80) and 100 % (M100) of *M. oleifera* forage (**a**), using steers and sheep as a source of inoculum (**b**).

Figure 1 shows the impact of the ruminal inoculum source and the percentage of MOL forage in the co-ensiling with maize on the kinetics of biogas production. The steers inoculum (STI) presented the lowest (*p* < 0.0001) delay in biogas production and the highest (*p* < 0.0001) rate and production of asymptotic biogas, which was reflected in a high (*p* < 0.0001) production of biogas by dry matter (DM) incubated and degraded, while the sheep inoculum (SHI) showed a completely opposite response. The inclusion of MOL decreased (*p* < 0.0001) the time in the lag phase at all levels, and at the M20 and M40 percentages it also reduced (*p* = 0.0171) the asymptotic biogas production. In addition, biogas production by DM incubated in M20 and M40 decreased (*p* = 0.0399), while in biogas by DM degraded this effect was observed (*p* ≤ 0.0235) in all silages with MOL. In the interaction, the inclusion of MOL did not affect the biogas production parameters with the STI, while with the SHI it only increased (*p* < 0.0001) the asymptotic biogas production. In the STI, it reduced (*p* = 0.0475) biogas production per DM incubated and degraded during the entire incubation period, except at 48 h per DM incubated. Contrary to this, with the SHI increased (*p* = 0.0475), biogas production by DM incubated at 24 and 48 h, as well as at 48 h by DM degraded, even though at 6 and 24 h it decreased (*p*< 0.0001; Table 2).

### 3.2. Ruminal Methane (CH_4_) Production

**Figure 2:**
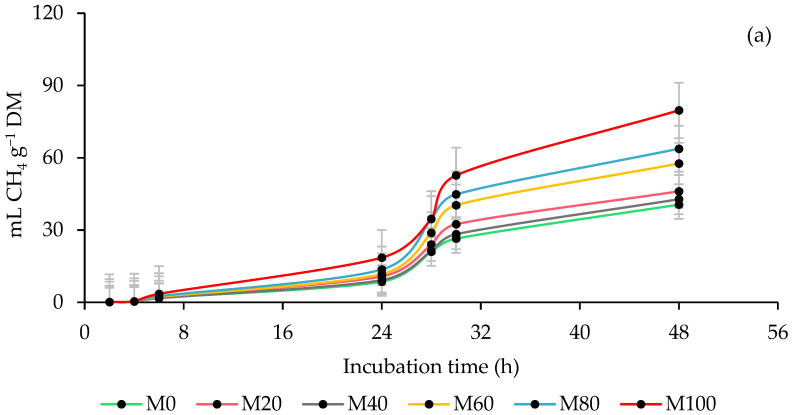

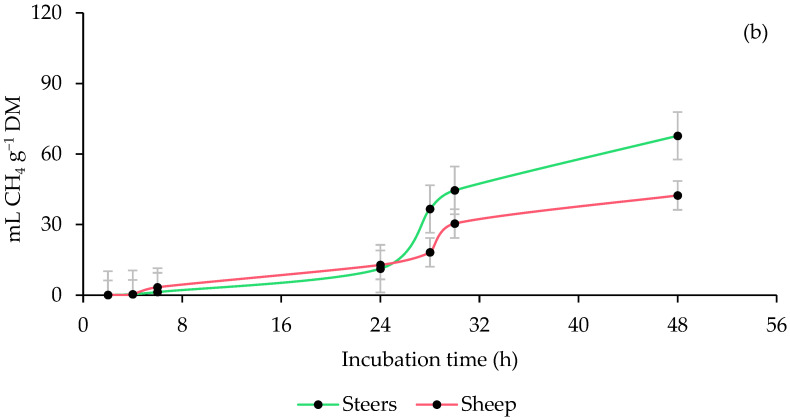
Kinetics of ruminal methane (CH_4_) production of co-ensiling of whole-plant maize (*Zea mays* L.) with 0 (M0), 20 (M20), 40 (M40), 60 (M60), 80 (M80) and 100 % (M100) of *M. oleifera* forage (**a**), using steers and sheep as a source of inoculum (**b**).

Figure 2 shows the impact of the rumen inoculum source and the percentage of MOL forage in the co-ensiling with maize on the kinetics of CH_4_ production. With STI reduced (*p* < 0.0001) time in lag phase and CH_4_ production rate and had the highest (*p* < 0.0001) asymptotic CH_4_ production compared to SHI. In the production of CH_4_, the STI increased (*p* = 0.0189) the production by DM incubated and degraded but without effect (*p* = 0.1585) at 24 h in the CH_4_ by DM incubated and a reduction (*p* = 0.0044) in the CH_4_ by DM degraded. The inclusion of MOL influenced (*p* ≤ 0.0259) at 24 and 48 h in the CH_4_ production by DM incubated, and at 6 and 24 h in the CH_4_ by DM degraded, and it was obtained that the silages with MOL produced more CH_4_ by DM incubated and degraded, except at 6 h due to DM degraded, since it decreased (*p* = 0.0259). In the interaction, it was found that the inclusion of MOL did not affect (*p* > 0.05) the parameters of CH_4_ production in both inoculums, and that it reduced (*p* = 0.0002) CH_4_ by DM degraded at 6 h with the STI, while that with the SHI the CH_4_ increased (*p* ≤ 0.0157) by DM incubated at 24 and 48 h, and by DM degraded at 6 h it decreased (*p* = 0.0002) and at 24 h it increased (*p* = 0.0307; Table 3).

In the proportion of CH_4_, it was observed that at 6 h it was lower (*p* < 0.0001) with the SHI and at 24 h with the STI, while in the CH_4_ per kg DM the lowest production (*p* ≤ 0.0006) was presented with the SHI at 6 and 48 h. In addition, with the inclusion of MOL, the proportion and production of CH_4_ per kg of DM increased (*p* ≤ 0.0120) at 24 and 48 h, and the interaction showed that this effect only occurs with SHI, which increased (*p* ≤ 0.0157) the ratio and CH_4_ per kg DM throughout the incubation by increasing the percentage of MOL, with no effect at 6 h on the CH_4_ ratio (Table 4). 

### 3.3. Ruminal Carbon Monoxide (CO) Production

**Figure 3:**
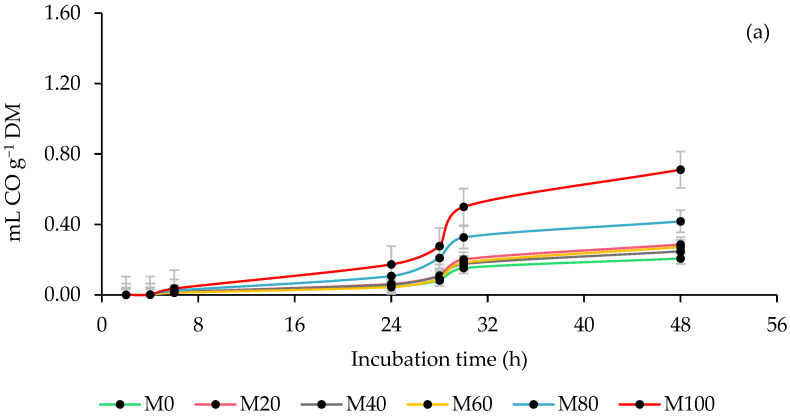

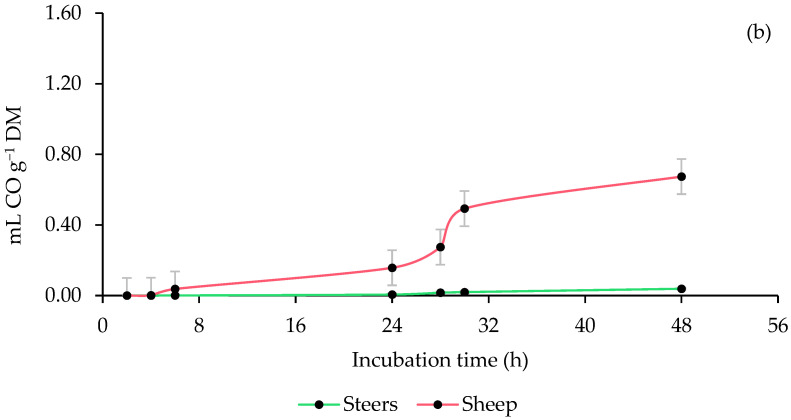
Kinetics of ruminal carbon monoxide (CO) production of co-ensiling of whole-plant maize (*Zea mays* L.) with 0 (M0), 20 (M20), 40 (M40), 60 (M60), 80 (M80) and 100 % (M100) of *M. oleifera* forage (**a**), using steers and sheep as a source of inoculum (**b**).

Figure 3 shows the impact of the rumen inoculum source and the percentage of MOL forage in the co-ensiling with maize on the kinetics of CO production. The STI presented the lowest values (*p* ≤ 0.0192) in the parameters and the production of CO by DM incubated and degraded throughout the incubation, while the SHI the highest. Excluding M40 and M60, all MOL inclusion percentages increased (*p* = 0.0094) asymptotic CO production, and at 48 h, all silages with MOL produced more (*p* ≤ 0.0006) CO per DM incubated and degraded, although in both cases there were slight variations (*p* ≤ 0.0331) at 6 and 24 h of incubation. In the interaction, it was found that the inclusion of MOL did not affect (*p* > 0.05) the parameters of CO production with the STI, but it did increase (*p* < 0.0001) the CO per DM incubated at 6 and 24 h and decreased (*p* ≤ 0.0051) CO per DM degraded throughout the incubation. In addition, with the SHI, the inclusion of MOL increased (*p* = 0.0094) the production of asymptotic CO and the production of CO by DM incubated and degraded (*p* ≤ 0.0051; Table 5).

### 3.4. Ruminal Hydrogen Sulfide (H_2_S) Production

**Figure 4:**
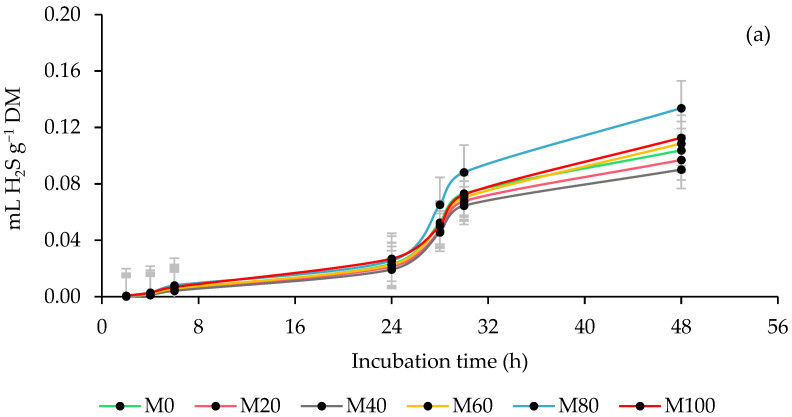

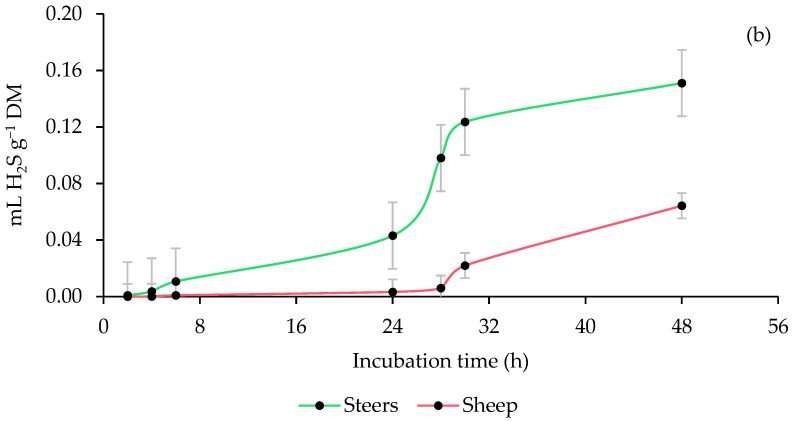
Kinetics of ruminal hydrogen sulfide (H_2_S) production of co-ensiling of whole-plant maize (*Zea mays* L.) with 0 (M0), 20 (M20), 40 (M40), 60 (M60), 80 (M80) and 100 % (M100) of *M. oleifera* forage (**a**), using steers and sheep as a source of inoculum (**b**).

Figure 4 shows the impact of the rumen inoculum source and the MOL forage percentage in co-ensiling with maize on the kinetics of H_2_S production. With the STI, the production of asymptotic H_2_S increased (*p* < 0.0001), the time in the lag phase decreased (*p* < 0.0001), and the production of H_2_S by DM incubated and degraded was greater (*p* < 0.0001), although at 48 h decreased (*p* < 0.0001) the amount of H_2_S per DM degraded, while with SHI the response was the opposite. The silages with MOL increased (*p* ≤ 0.0157) the production of H_2_S by DM incubated at 6 and 48 h, and at 6 and 24 h by DM degraded, except the M20 and M40 levels that produced less (*p* ≤ 0.0157) H_2_S in all incubations. However, the interaction showed that, with the STI and the inclusion of MOL, the production of H_2_S by DM incubated and degraded was reduced (*p* ≤ 0.0237) during the entire incubation, except at 24 h for incubated MS (*p* > 0.05). Contrary to this, with the SHI, the production of H_2_S only decreased (*p* ≤ 0.0015) at 6 h and increased (*p* ≤ 0.0046) the remaining incubation both due to DM incubated and degraded (Table 6).

### 3.5. Ruminal Fermentation Characteristics and CH_4_ Conversion Efficiency

STI increased (*p* < 0.0001) dry matter degraded (DMD), short-chain fatty acids (SCFA), and metabolizable energy (ME) but decreased (*p* < 0.0001) pH and CH_4_ production per unit SCFA and ME, so it turned out to be more efficient. The inclusion of MOL gradually increased (*p* = 0.0063) the DMD, and in the case of the M80 and M100 percentages, the SCFA and ME also increased (*p* ≤ 0.0064). Despite this, the silages with MOL were not very efficient due to their high (*p* ≤ 0.0006) CH_4_ production per unit of SCFA, ME, and OM. In the interaction it was observed that, with STI, the inclusion of MOL increased (*p* ≤ 0.0237) the pH and the DMD, and decreased the SCFA (*p* = 0.0001) and ME (*p* = 0.0128), without affecting (*p* > 0.05) the production of CH_4_ per unit of AGCC, ME, and OM. Meanwhile, with SHI increased DMD (*p* = 0.0127), SCFA (*p* = 0.0001), and ME (*p* = 0.0128), as well as CH_4_ production (*p* ≤ 0.0023) per unit of SCFA, ME, and OM, which led to inefficiency in CH_4_ conversion (Table 7).

## 4. Discussion

### 4.1. Ruminal Biogas Production

Although it has been reported that the food ingested by the donor animals before the collection of the rumen fluid influences the microbiota of the inoculum [56], in the present study, it was observed that the fermentation patterns were similar during incubation between the steers (STI) and sheep (SHI) inoculum, and when comparing the biogas production of both inoculums, the highest production was obtained with the STI. In this regard, it has been reported that, in ruminants (sheep, goats, cattle, and buffalo), it is possible that the microbial metabolic pathways may be qualitatively similar but quantitatively different between species [36,57], and that this is due to variations in the diversity and microbial load of each ruminant [58]. Likewise, during the ruminal digestion of the feed, the microorganisms first break down and ferment the carbohydrates, and as a product of this fermentation they release short-chain fatty acids, mainly acetate, propionate, and butyrate, in addition to biogas [59]. On the other hand, the percentages of inclusion of *Moringa oleifera* (MOL) showed a similar fermentation pattern with slight variations in the amount of biogas, which is presumably due to the observed balance between cellulose and hemicellulose, since it has been reported that hemicellulose increases biogas production, while cellulose decreases it, and in this evaluation said relationship was maintained between 0.9778 and 1.1768 (Table 1). In addition, although the inclusion of MOL increased the percentage of protein and ethereal extract, the gas produced by the fermentation of these nutrients is lower compared to carbohydrates [60]. Therefore, the contribution of gas to the total production is low and not may be the cause of this effect. On the other side, the rumen inoculum source influenced the effect of MOL inclusion on biogas production, which is why it differed between inoculums. With the STI, the inclusion of MOL decreased the production of biogas and with the SHI it increased it, except at 6 and 24 h due to DM degraded. This indicates that the SHI possibly presented low microbial activity, since although the inclusion of MOL decreased the time in the lag phase, it continued to be longer compared to the STI, and this reduced the fermentation time (Table 1). 

### 4.2. Ruminal Methane (CH_4_) Production

With the STI, CH_4_ production was higher compared to the SHI, and this can be attributed to a higher population of methanogen microbes in the STI. These microorganisms use the hydrogen (H_2_) produced from carbon monoxide (CO) to reduce carbon dioxide (CO_2_) to CH_4_ [61], a process known as methanogenesis, which represents the main metabolic route used as a sink source of H_2_ in the rumen [4]. In addition, the CO production obtained with the sheep inoculum supports the assumption that the ruminal methanogenic population influenced CH_4_ production, since although it had a high CO production with the SHI (which translates into a higher availability of CO_2_ and H_2_), the production of CH_4_ was low compared to that obtained with the bovine inoculum. In the silages, the inclusion of MOL gradually reduced the proportion of maize and hemicellulose and increased CH_4_ production, despite the positive correlation between hemicellulose and CH_4_ production. It is believed that this inverse effect may be related to the concentration of water-soluble carbohydrates in maize plants, especially starch, since it generates an unfavorable environment (lower pH and acetate:propionate ratio) for methanogens and protozoa, which suppresses their growth and consequently reduces CH_4_ production [62,63]. In addition, it is not ruled out that in some silages the production of H_2_S could have caused a competition for H_2_ between sulfate-reducing bacteria (SRB) and methanogens [64], since H_2_S provides an alternative pathway sink of H_2_ and consequently reduces methanogenesis [65].

### 4.3. Ruminal Carbon Monoxide (CO) Production

CO is a gas that is produced during the degradation of organic matter (OM) by the ruminal microbiota [66]. Therefore, its production is attributed to microbial activity and the fermentative capacity of microorganisms and rumens of each inoculum. In addition to the above, it is important to mention that CO is oxidized in contact with water (H_2_O) to form CO_2_ and H_2_, which are used by methanogens for the formation of CH_4_ [67]. Therefore, it is believed that the low production of CO with STI is a consequence of rapid oxidation and the formation of other gases, which was reflected in a higher production of CH_4_ and H_2_S compared to SHI. As already mentioned above, CO is produced during OM degradation, but it was observed that the inclusion of MOL gradually reduced OM and increased CO production, so it can be assumed that production is more influenced by the type and concentration of OM phytoconstituents than by the amount of OM. In this regard, it has been reported that CH_4_ is a natural source of CO [68], for which the statement is consistent if it is considered that the production of CH_4_ depends on the chemical composition of the forage, especially the quality of the fiber.

### 4.4. Ruminal Hydrogen Sulfide (H_2_S) Production

H_2_S is a component of biogas, and its production in the rumen can cause toxicity in animals and even metabolic alterations that lead to diseases [69]. H_2_S is produced during ruminal feed fermentation by SRB that reduce sulfur (S) to H_2_S [59], so the discrepancy in H_2_S production between ruminal inoculum sources is considered to be the result of a high population of SRB in the bovine inoculum, since the same silages were evaluated in both inoculums. On the other hand, the variations between the silages are attributed to the chemical composition, especially to the concentrations that they could have of S, and although it is part of the methionine and cysteine, amino acids necessary for protein synthesis, the excess (>3 g S kg^−1^ DM) of this mineral leads to a higher concentration of H_2_S in the rumen [70], and being rapidly absorbed causes animals to be sensitive to the toxicity of this gas [65].

### 4.5. Ruminal Fermentation Characteristics and CH_4_ Conversion Efficiency

The differences in the characteristics of fermentation and methane conversion efficiency between sources of ruminal inoculum are related to the diversity and quantity of ruminal microorganisms in the inoculum of each species [57], which consequently reflects on the microbial activity and the fermentative capacity of the ruminal microbiota, as well as the final products of fermentation [58,71]. On the other hand, the observed increase in pH with the inclusion of MOL in the STI could have occurred due to the reduction in the percentage of maize, since this led to a lower availability of fast-fermenting carbohydrates such as starch, which generally lowers the pH [72,73]. Similarly, dry matter degraded (DMD) increased in both inoculum, which is desirable from a nutritional point of view, and is attributed to the pH, which could have generated a more favorable environment for microbial proliferation, especially cellulolytic bacteria, and to the protein available for microbial growth in each silage [29,74], which, when in balance, promotes efficient feed degradation. Although short-chain fatty acids (SCFA) and metabolizable energy (ME) are positively related to DMD, the inclusion of MOL decreased SCFA and ME with the STI, while with the SHI it increased them but was less efficient due to the gradual increase of CH_4_ per unit of SCFA, EM, and MO in comparison with the STI, where there was no difference in the efficiency between silages. The lack of difference between silages with the STI can be attributed to the production of H_2_S, since it could have reduced methanogenesis [66], while with the SHI it could be caused by a higher production of acetate and butyrate, which release CO_2_ and H_2_ and, as these gases are more available in the rumen, CH_4_ production increases [75].

## 5. Conclusions

The co-ensiling of whole-plants maize with increasing percentages of *Moringa oleifera* forage had a different impact on biogas production kinetics and rumen fermentation with the evaluated inoculum sources, and although the amount of biogas was higher with the inoculum steer and lower with the sheep inoculum, the inclusion of *M. oleifera* increased the DMD with both inoculums. In the case of the steer inoculum, the increase in degradability did not cause a negative impact on the CH_4_ ratio and CH_4_ production per unit of SCFA, ME, and OM. In addition, with this inoculum, the inclusion of *M. oleifera* reduced biogas, CO, and H_2_S by degraded DM in most of the silages, without affecting CH_4_ production. Despite the above, it is possible that, with the increase in DMD, the productive response in both species will improve, and that biogas production per unit of meat or milk will decrease, and if so, the co-ensiling of maize with *M. oleifera* can be considered as an option to reduce the impact of livestock on the environment.

## Figures and Tables

**Table 1:** Chemical composition (%, on DM basis) of the co-ensiling ^1^ of whole-plants maize (*Zea mays* L.) with increasing percentages of *Moringa oleifera* forage, at 120 days of fermentation.

Percentage of Moringa ^2^	Component ^3^							
	OM	CP	EE	HEM	CEL	LIG	NSC	TC
M0	94.86	8.23	1.58	26.13	30.14	4.46	24.32	85.05
M20	94.51	9.21	1.83	26.64	29.41	4.69	22.73	83.47
M40	93.91	11.18	1.94	24.36	26.95	5.20	24.28	80.79
M60	92.77	13.55	2.02	22.85	23.97	5.76	24.61	77.20
M80	92.30	17.37	2.89	21.35	20.88	5.93	23.88	72.04
M100	91.01	20.12	3.92	15.77	18.55	6.49	26.16	66.97

^1^ The pH of the silages ranged between 3.64 and 4.25. ^2^ Percentage inclusion of *Moringa oleifera* forage in the co-ensiling with whole-plants maize (*Zea mays* L.): 0 (M0), 20 (M20), 40 (M40), 60 (M60), 80 (M80) and 100% (M100). ^3^ OM: organic matter; CP: crude protein; EE: ether extract; HEM: hemicellulose; CEL: cellulose; LIG: acid detergent lignin; NSC: non-structural carbohydrates; TC: total carbohydrates.

**Table 2:** Parameters and in vitro ruminal biogas production of the co-ensiling of whole-plants maize (*Zea mays* L.) with increasing percentages of *Moringa oleifera* forage, using steers and sheep as inoculum sources.

Inoculum Source Ruminal (ISR)	Percentage of Moringa (PMOL) ^1^	Biogas Production								
		Parameters ^2^			mL Gas g^−1^ DM Incubated			mL Gas g^−1^ DM Degraded		
		*b*	*c*	*Lag*	6 h	24 h	48 h	6 h	24 h	48 h
Steers	M0	396.07	0.0283	2.17	125.55	283.41	390.61	492.08	1110.97	1530.14
	M20	319.97	0.0293	1.75	108.30	239.50	318.29	282.92	625.58	831.07
	M40	320.87	0.0284	1.76	107.92	236.53	317.76	316.87	694.44	932.90
	M60	374.83	0.0294	2.05	118.15	254.71	367.81	359.77	775.63	1120.00
	M80	457.23	0.0282	2.50	135.64	276.50	439.74	452.40	923.99	1474.65
	M100	370.57	0.0278	2.03	134.94	275.91	366.44	451.39	922.68	1225.77
	SEM ^3^	27.04	0.0007	0.15	3.90	9.31	24.02	18.05	45.44	94.30
	*p*-value									
	Linear	0.0698	0.3631	0.0698	0.0087	0.0059	0.0547	<0.0001	<0.0001	0.0002
	Quadratic	0.7113	0.2733	0.7114	0.0027	0.2289	0.6909	0.0136	0.3476	0.7026
Sheep	M0	166.20	0.0252	10.30	69.20	125.32	163.12	320.59	580.46	755.36
	M20	227.40	0.0253	7.98	79.69	166.93	221.35	363.17	760.56	1008.35
	M40	180.50	0.0235	9.71	66.98	128.13	174.40	290.67	556.04	756.98
	M60	221.25	0.0238	7.33	71.21	149.83	212.62	288.82	607.46	862.17
	M80	237.00	0.0251	4.80	64.65	156.08	226.91	227.27	548.61	797.80
	M100	272.55	0.0267	2.87	65.29	181.17	262.48	204.04	567.07	821.12
	SEM ^3^	11.71	0.0010	0.47	3.12	10.33	12.14	15.37	40.81	49.28
	*p*-value									
	Linear	0.0101	0.9155	0.0128	0.0551	0.0293	0.0146	0.0978	0.0206	0.0110
	Quadratic	0.0019	0.2557	<0.0001	0.0536	0.0325	0.0032	0.0003	0.0839	0.3530
Pooled SEM ^3^		25.31	0.0008	0.28	3.90	10.04	22.73	18.27	46.49	89.46
*p*-value										
ISR		<0.0001	<0.0001	<0.0001	<0.0001	<0.0001	<0.0001	<0.0001	<0.0001	<0.0001
PMOL		0.0171	0.5988	<0.0001	0.0399	0.0064	0.0157	0.0006	0.0049	0.0235
Linear		0.7730	0.5103	0.0001	0.3996	0.9104	0.7614	0.0003	0.0043	0.0232
Quadratic		0.0604	0.7539	<0.0001	0.2088	0.0111	0.0523	0.0319	0.5522	0.9215
ISR × PMOL		0.0571	0.1613	<0.0001	0.0002	0.0128	0.0475	<0.0001	<0.0001	0.0006

^1^ Percentage inclusion of *Moringa oleifera* forage in the co-ensiling with whole-plants maize (*Zea mays* L.): 0 (M0), 20 (M20), 40 (M40), 60 (M60), 80 (M80) and 100% (M100). ^2^ *b* is the asymptotic biogas production (mL gas g^−1^ DM); *c* is the rate biogas production (mL gas h^−1^); *Lag* is the initial delay before biogas production begins (h). ^3^ SEM, standard error of the mean.

**Table 3:** Parameters and in vitro ruminal methane (CH_4_) production of the co-ensiling of whole-plants maize (*Zea mays* L.) with increasing percentages of *Moringa oleifera* forage, using steers and sheep as inoculum sources.

Inoculum Source Ruminal (ISR)	Percentage of Moringa (PMOL) ^1^	CH_4_ Production								
		Parameters ^2^			mL Gas g^−1^ DM Incubated			mL Gas g^−1^ DM Degraded		
		*b*	*c*	*Lag*	6 h	24 h	48 h	6 h	24 h	48 h
Steers	M0	58.32	0.0826	10.10	1.23	9.86	58.45	4.83	38.36	226.93
	M20	61.35	0.0919	10.63	1.14	10.21	61.62	2.96	26.65	160.99
	M40	62.59	0.0856	10.84	1.19	10.93	62.74	3.48	32.09	184.27
	M60	76.95	0.0944	13.33	1.34	12.10	77.19	4.08	36.81	235.10
	M80	82.98	0.0960	14.37	1.58	13.06	83.63	5.28	43.80	280.55
	M100	62.50	0.0895	10.83	1.35	11.47	62.84	4.52	38.33	210.08
	SEM ^3^	9.53	0.0042	1.65	0.07	1.13	9.60	0.25	4.15	35.24
	*p*-value									
	Linear	0.8260	0.1404	0.8261	0.3616	0.8264	0.8194	0.0002	0.0690	0.2104
	Quadratic	0.8228	0.6704	0.8229	0.0801	0.3173	0.8152	0.0661	0.2740	0.7152
Sheep	M0	22.68	0.0997	20.80	0.44	7.27	22.63	2.04	33.85	105.30
	M20	30.79	0.1051	20.86	0.50	11.29	30.50	2.27	51.37	138.80
	M40	22.95	0.1003	21.08	0.33	7.46	22.82	1.45	32.44	99.16
	M60	38.25	0.1205	21.71	0.36	11.32	38.02	1.44	45.93	153.59
	M80	9.12	0.1123	21.07	0.32	14.17	43.86	1.14	49.70	154.42
	M100	10.05	0.1057	22.05	0.33	25.63	96.49	1.02	80.45	303.04
	SEM ^3^	4.60	0.0126	0.73	0.06	2.96	11.12	0.31	10.92	39.94
	*p*-value									
	Linear	0.2590	0.7701	0.9553	0.5395	0.3734	0.6347	0.6173	0.2999	0.5748
	Quadratic	0.0252	0.8390	0.2217	0.1174	0.0041	0.0022	0.0249	0.0300	0.0101
Pooled SEM ^3^		8.98	0.0076	1.55	0.07	1.86	10.49	0.28	6.85	38.25
*p*-value										
ISR		<0.0001	0.0011	<0.0001	<0.0001	0.1585	0.0006	<0.0001	0.0044	0.0189
PMOL		0.3128	0.2902	0.6007	0.2638	0.0005	0.0120	0.0259	0.0144	0.0598
Linear		0.5451	0.3473	0.8531	0.7941	0.2561	0.6070	0.0103	0.6783	0.6780
Quadratic		0.3819	0.6829	0.5417	0.8260	<0.0001	0.0009	0.3150	0.0018	0.0084
ISR × PMOL		0.2280	0.9685	0.6991	0.0108	0.0021	0.0157	0.0002	0.0307	0.0876

^1^ Percentage inclusion of *Moringa oleifera* forage in the co-ensiling with whole-plants maize (*Zea mays* L.): 0 (M0), 20 (M20), 40 (M40), 60 (M60), 80 (M80) and 100% (M100). ^2^ *b* is the asymptotic CH_4_ production (mL gas g^−1^ DM); *c* is the rate CH_4_ production (mL gas h^−1^); *Lag* is the initial delay before CH_4_ production begins (h). ^3^ SEM, standard error of the mean.

**Table 4:** In vitro ruminal methane (CH_4_) production based on biogas and kg DM of the co-ensiling of whole-plants maize (*Zea mays* L.) with increasing percentages of *Moringa oleifera* forage, using steers and sheep as inoculum sources.

Inoculum Source Ruminal (ISR)	Percentage of Moringa (PMOL) ^1^	CH_4_ Production					
		mL CH_4_ 100 mL^−1^ Biogas			g CH_4_ kg^−1^ DM		
		6 h	24 h	48 h	6 h	24 h	48 h
Steers	M0	0.98	3.53	15.17	5.73	45.83	271.79
	M20	1.05	4.27	19.35	5.28	47.49	286.52
	M40	1.10	4.62	19.68	5.52	50.80	291.75
	M60	1.13	4.75	20.92	6.23	56.25	358.95
	M80	1.17	4.72	18.92	7.36	60.72	388.88
	M100	1.00	4.13	17.08	6.30	53.35	292.22
	SEM ^2^	0.04	0.39	2.00	0.34	5.23	44.63
	*p*-value						
	Linear	0.3061	0.2046	0.1649	0.3617	0.8265	0.8194
	Quadratic	0.7629	0.6310	0.9442	0.0801	0.3173	0.8152
Sheep	M0	0.63	5.69	13.69	2.04	33.80	105.24
	M20	0.63	6.75	13.75	2.31	52.52	141.83
	M40	0.50	5.88	13.13	1.56	34.71	106.10
	M60	0.50	7.56	17.81	1.66	52.65	176.80
	M80	0.50	9.06	19.31	1.50	65.88	203.97
	M100	0.50	13.94	36.19	1.52	119.16	448.70
	SEM ^2^	0.07	1.23	3.20	0.29	13.76	51.73
	*p*-value						
	Linear	1.0000	0.5644	0.9894	0.5396	0.3734	0.6347
	Quadratic	0.2070	0.0022	0.0012	0.1175	0.0041	0.0022
Pooled SEM ^2^		0.06	0.74	2.47	0.34	8.64	48.78
*p*-value							
ISR		<0.0001	<0.0001	0.7525	<0.0001	0.1585	0.0006
PMOL		0.6616	0.0002	0.0021	0.2638	0.0005	0.0120
Linear		0.5548	0.2401	0.4045	0.7940	0.2561	0.6070
Quadratic		0.1570	<0.0001	<0.0001	0.8258	<0.0001	0.0009
ISR × PMOL		0.0719	0.0002	0.0007	0.0108	0.0021	0.0157

^1^ Percentage inclusion of *Moringa oleifera* forage in the co-ensiling with whole-plants maize (*Zea mays* L.): 0 (M0), 20 (M20), 40 (M40), 60 (M60), 80 (M80) and 100% (M100). ^2^ SEM, standard error of the mean.

**Table 5:** Parameters and in vitro ruminal carbon monoxide (CO) production of the co-ensiling of whole-plants maize (*Zea mays* L.) with increasing percentages of *Moringa oleifera* forage, using steers and sheep as inoculum sources.

Inoculum Source Ruminal (ISR)	Percentage of Moringa (PMOL) ^1^				CO Production					
		Parameters ^2^			mL CO g^−1^ DM Incubated			mL CO g^−1^ DM Degraded		
		*b*	*c*	*Lag*	6 h	24 h	48 h	6 h	24 h	48 h
Steers	M0	0.02153	0.00009	0.00003	0.00050	0.00546	0.04038	0.00194	0.02139	0.15822
	M20	0.03350	0.00008	0.00004	0.00054	0.00534	0.04067	0.00140	0.01395	0.10607
	M40	0.03847	0.00008	0.00203	0.00054	0.00549	0.03604	0.00158	0.01613	0.10575
	M60	0.01837	0.00006	0.00002	0.00087	0.00459	0.03231	0.00264	0.01397	0.09841
	M80	0.02593	0.00006	0.00003	0.00099	0.00589	0.04164	0.00333	0.01960	0.13948
	M100	0.03630	0.00010	0.00005	0.00130	0.00850	0.04075	0.00436	0.02851	0.13652
	SEM ^3^	0.00868	0.00002	0.00081	0.00006	0.00062	0.00306	0.00024	0.00208	0.01112
	*p*-value									
	Linear	0.6072	0.0199	0.6076	0.3770	0.1488	0.0006	0.0053	0.0005	<0.0001
	Quadratic	0.4452	0.4909	0.4452	0.0019	0.0877	0.0122	0.0025	0.1022	0.0267
Sheep	M0	5.47365	0.00943	9.70000	0.00169	0.08639	0.37269	0.00784	0.40196	1.73135
	M20	9.29490	0.00631	7.65240	0.00203	0.11812	0.53105	0.00926	0.53562	2.41206
	M40	2.24535	0.00024	5.35000	0.00165	0.11192	0.45795	0.00714	0.48624	1.98784
	M60	2.97805	0.00076	4.50000	0.00200	0.08380	0.51019	0.00808	0.34433	2.06793
	M80	6.35000	0.00031	4.20000	0.00168	0.20790	0.79209	0.00591	0.73058	2.79043
	M100	25.95985	0.01201	6.65000	0.00114	0.33702	1.38065	0.00358	1.05746	4.32665
	SEM ^3^	5.69736	0.00609	2.80410	0.00017	0.04020	0.12264	0.00066	0.15639	0.46880
	*p*-value									
	Linear	0.6521	0.7297	0.6241	0.2124	0.5970	0.3964	0.1783	0.5678	0.3441
	Quadratic	0.0374	0.5994	0.5767	0.0144	0.0031	0.0008	0.0008	0.0218	0.0077
Pooled SEM ^3^		2.98757	0.00319	1.47041	0.00011	0.02109	0.06437	0.00041	0.08203	0.24604
*p*-value										
ISR		<0.0001	0.0192	<0.0001	<0.0001	<0.0001	<0.0001	<0.0001	<0.0001	<0.0001
PMOL		0.0094	0.3091	0.4406	0.0331	<0.0001	<0.0001	0.0206	0.0037	0.0006
Linear		0.5313	0.6321	0.4973	0.0946	0.4656	0.2359	0.2979	0.4539	0.2199
Quadratic		0.0022	0.4652	0.4389	0.7268	<0.0001	<0.0001	0.005	0.0005	<0.0001
ISR × PMOL		0.0094	0.3144	0.4404	<0.0001	<0.0001	<0.0001	<0.0001	0.0051	0.0007

^1^ Percentage inclusion of *Moringa oleifera* forage in the co-ensiling with whole-plants maize (*Zea mays* L.): 0 (M0), 20 (M20), 40 (M40), 60 (M60), 80 (M80) and 100% (M100). ^2^ *b* is the asymptotic CO production (ppm gas g^−1^ DM); *c* is the rate CO production (ppm gas h^−1^); *Lag* is the initial delay before CO production begins (h). ^3^ SEM, standard error of the mean.

**Table 6:** Parameters and in vitro ruminal hydrogen sulfide (H_2_S) production of the co-ensiling of whole-plants maize (*Zea mays* L.) with increasing percentages of *Moringa oleifera* forage, using steers and sheep as inoculum sources.

Inoculum Source Ruminal (ISR)	Percentage of Moringa (PMOL) ^1^	H_2_S Production								
		Parameters ^2^			mL H_2_S g^−1^ DM Incubated			mL H_2_S g^−1^ DM Degraded		
		*b*	*c*	*Lag*	6 h	24 h	48 h	6 h	24 h	48 h
Steers	M0	0.10800	0.00021	0.00080	0.00911	0.04526	0.1669	0.03578	0.17736	0.13267
	M20	0.11613	0.00015	0.00086	0.00797	0.03823	0.1257	0.02084	0.09992	0.03632
	M40	0.11287	0.00015	0.00084	0.00790	0.03645	0.1262	0.02320	0.10704	0.04467
	M60	0.10877	0.00019	0.00081	0.01093	0.04289	0.1509	0.03331	0.13054	0.10073
	M80	0.14127	0.00022	0.00105	0.01497	0.04665	0.1781	0.04995	0.15621	0.11396
	M100	0.12260	0.00020	0.00091	0.01281	0.04908	0.1586	0.04283	0.16389	0.11687
	SEM ^3^	0.01089	0.00002	0.00008	0.00088	0.00322	0.0031	0.00311	0.01165	0.01047
	*p*-value									
	Linear	0.6072	0.0199	0.6076	0.3770	0.1488	0.0006	0.0053	0.0005	<0.0001
	Quadratic	0.4452	0.4909	0.4452	0.0019	0.0877	0.0122	0.0025	0.1022	0.0267
Sheep	M0	0.04160	0.12475	7.08305	0.00008	0.00107	0.04079	0.00036	0.00490	0.12728
	M20	0.08320	0.00003	5.20000	0.00006	0.00458	0.06825	0.00029	0.02084	0.20312
	M40	0.03077	0.00014	6.00000	0.00006	0.00218	0.05403	0.00026	0.00945	0.15613
	M60	0.02450	0.00005	7.20000	0.00007	0.00228	0.06635	0.00030	0.00928	0.17636
	M80	0.03675	0.00048	8.30000	0.00007	0.00460	0.08928	0.00024	0.01626	0.20082
	M100	0.02200	0.00010	8.35000	0.00002	0.00483	0.06676	0.00006	0.01521	0.14296
	SEM ^3^	0.02409	0.04636	1.36597	0.00001	0.00100	0.00780	0.00004	0.00360	0.02784
	*p*-value									
	Linear	0.2679	0.1058	0.3673	0.3857	0.0465	0.1716	0.2858	0.0204	0.1024
	Quadratic	0.2200	0.3147	0.2349	0.0045	0.1505	0.3613	0.0014	0.6159	0.5386
Pooled SEM ^3^		0.01605	0.02431	0.71628	0.00080	0.00297	0.00494	0.00282	0.01075	0.01742
*p*-value										
ISR		<0.0001	0.1588	<0.0001	<0.0001	<0.0001	<0.0001	<0.0001	<0.0001	<0.0001
PMOL		0.3394	0.1035	0.2377	0.0016	0.1777	0.0067	0.0005	0.0157	0.0828
Linear		0.1405	0.0199	0.2074	0.4816	0.5644	0.7686	0.0165	0.0107	0.5655
Quadratic		0.2991	0.1584	0.0935	0.0072	0.0876	0.0287	0.0096	0.1578	0.7420
ISR × PMOL		0.2999	0.1037	0.2378	0.0015	0.3199	0.0237	0.0005	0.0046	0.0018

^1^ Percentage inclusion of *Moringa oleifera* forage in the co-ensiling with whole-plants maize (*Zea mays* L.): 0 (M0), 20 (M20), 40 (M40), 60 (M60), 80 (M80) and 100% (M100). ^2^ *b* is the asymptotic H_2_S production (ppm gas g^−1^ DM); *c* is the rate H_2_S production (ppm gas h^−1^); *Lag* is the initial delay before H_2_S production begins (h). ^3^ SEM, standard error of the mean.

**Table 7:** Fermentation characteristics and CH_4_ conversion efficiency of the co-ensiling of whole-plants maize (*Zea mays* L.) with increasing percentages of *Moringa oleifera* forage, using steers and sheep as inoculum sources.

Inoculum Source Ruminal (ISR)	Percentage of Moringa (PMOL) ^1^	Fermentation Characteristics ^2^				CH_4_ Conversion Efficiency ^3^		
		pH	DMD	SCFA	ME	CH_4_:SCFA	CH_4_:ME	CH_4_:OM
Steers	M0	6.33	60.58	6.27	8.82	46.27	5.22	10.94
	M20	6.36	75.53	5.30	8.32	55.90	5.71	11.34
	M40	6.46	72.01	5.23	8.29	60.49	6.13	12.13
	M60	6.55	75.52	5.63	8.49	62.22	6.62	13.43
	M80	6.55	74.14	6.12	8.74	61.76	6.94	14.49
	M100	6.67	81.05	6.10	8.73	54.12	6.09	12.73
	SEM ^4^	0.07	1.430	0.211	0.112	5.060	0.581	1.250
	*p*-value							
	Linear	0.7778	<0.0001	0.0059	0.0060	0.2032	0.5642	0.8264
	Quadratic	0.0015	<0.0001	0.2291	0.2296	0.6327	0.3962	0.3173
Sheep	M0	7.09	43.25	2.76	5.03	74.78	6.67	7.85
	M20	7.06	43.89	3.69	5.51	88.59	9.53	12.20
	M40	7.06	46.07	2.82	5.07	77.25	6.87	8.06
	M60	7.03	49.32	3.31	5.31	99.32	9.91	12.23
	M80	7.02	56.90	3.44	5.38	118.99	12.23	15.31
	M100	6.98	64.14	4.00	5.67	182.84	20.83	27.69
	SEM^4^	0.041	0.992	0.230	0.122	16.151	2.163	3.202
	*p*-value							
	Linear	0.6207	0.6648	0.0292	0.0292	0.5676	0.3853	0.3734
	Quadratic	0.1051	<0.0001	0.0325	0.0325	0.0022	0.0029	0.0041
Pooled SEM ^4^		0.06	1.40	0.22	0.11	9.64	1.25	2.02
*p*-value								
ISR		<0.0001	<0.0001	<0.0001	<0.0001	<0.0001	<0.0001	0.2548
PMOL		0.3955	0.0063	<0.0001	0.0064	0.0002	0.0001	0.0006
Linear		0.9793	<0.0001	0.9115	0.9108	0.2418	0.1994	0.2589
Quadratic		0.0491	<0.0001	0.0111	0.0111	<0.0001	<0.0001	<0.0001
ISR × PMOL		0.0237	0.0127	0.0001	0.0128	0.0002	0.0003	0.0023

^1^ Percentage inclusion of *Moringa oleifera* forage in the co-ensiling with whole-plants maize (*Zea mays* L.): 0 (M0), 20 (M20), 40 (M40), 60 (M60), 80 (M80) and 100% (M100). ^2^ pH is ruminal pH; DMD is dry matter degradability (%); SCFA is short-chain fatty acids (mmol g^−1^ DM) at 24 h of incubation; ME is the metabolizable energy (MJ kg^−1^ DM) at 24 h of incubation. ^3^ CH_4_:SCFA is methane:short-chain fatty acid ratio (mmol mmol^−1^) at 24 h of incubation; CH_4_:ME is methane:metabolizable energy ratio (g MJ^−1^) at 24 h of incubation; CH_4_:OM is methane:organic matter ratio (mL g^−1^). ^4^ SEM, standard error of the mean.

## Data Availability

References were carefully checked, and changes were made without modify the format.
